# Evaluating Neutralizing Antibody Titers by Recombinant Feline Calicivirus with Heterologous Capsid Protein VP1

**DOI:** 10.3390/ani16081237

**Published:** 2026-04-17

**Authors:** Yang Wang, Wei Lin, Yue Zhang, Hongling He, Yueming Wang, Saisai Li, Qiuyuan Zhang, Shile Huang, Jun Luo, Xiaofeng Guo

**Affiliations:** 1 College of Veterinary Medicine, South China Agricultural University, Guangzhou 510642, China; 2 Department of Biochemistry and Molecular Biology, Louisiana State University Health Sciences Center, 1501 Kings Highway, Shreveport, LA 71130-3932, USA; 3 Department of Hematology and Oncology, Louisiana State University Health Sciences Center, 1501 Kings Highway, Shreveport, LA 71130-3932, USA; 4 Feist-Weiller Cancer Center, Louisiana State University Health Sciences Center, 1501 Kings Highway, Shreveport, LA 71130-3932, USA

**Keywords:** feline calicivirus, infectious clone, *VP1* gene, growth kinetics, neutralizing antibody

## Abstract

Feline calicivirus (FCV) is a major pathogen that threatens feline health worldwide. Its global prevalence, extensive genetic variability, and limited cross-protection among strains present significant challenges for vaccine development. In this study, an infectious clone of the FCV-GDJM202201 strain was constructed using the eukaryotic expression plasmid pcDNA3.1 under the control of the cytomegalovirus (CMV) promoter. The rescued virus, rGDJM-A4822T, exhibited growth kinetics comparable to those of the parental strain in vitro. Subsequently, two recombinant viruses, rGDJM-VP1_JL_ and rGDJM-VP1_SH_, were generated by replacing the *VP1* gene in the GDJM202201 backbone with those from heterologous FCV strains. Notably, these recombinant viruses exhibited reduced viral titers compared to rGDJM-A4822T. Finally, neutralization assays revealed differential neutralizing antibody titers among the recombinant FCVs, with rGDJM-A4822T inducing higher neutralizing antibody titers and cross-neutralizing activity. Collectively, this study establishes an FCV infectious clone that can be used to rescue recombinant viruses carrying heterologous VP1 proteins and to evaluate neutralizing antibody responses.

## Simple Summary

Feline calicivirus (FCV) is a common viral pathogen that poses a persistent threat to cat health worldwide. High genetic diversity and limited cross-protection among FCV strains complicate vaccine development. In this study, we established a reverse genetics system that enables precise manipulation of the FCV genome. Using this platform, we generated recombinant FCVs by replacing the original capsid protein with those derived from different FCV strains and evaluated their growth properties and neutralizing antibody responses. This system provides a valuable tool for investigating FCV gene function and assessing immune responses, thereby supporting the development of more effective vaccines.

## 1. Introduction

Feline calicivirus (FCV) is a positive-sense, single-stranded RNA virus belonging to the genus *Vesivirus* within the family *Caliciviridae*, which also includes clinically important pathogens such as human norovirus, rabbit hemorrhagic disease virus, and Sapporo virus [1,2]. Common clinical manifestations of FCV infection in cats include oral ulcers, gingivostomatitis, and upper respiratory symptoms, while kittens frequently develop more severe symptoms that may progress to fatal pneumonia and lameness [3,4]. Additionally, a variant known as virulent systemic FCV can cause jaundice, edema, and high mortality [5]. In the absence of effective antiviral therapies, vaccination remains the primary strategy for controlling FCV infection to alleviate clinical signs and reduce virus shedding; both attenuated and inactivated vaccines are commercially available [6,7]. However, the limited proofreading capability of the viral polymerase protein results in extensive genetic variability of the FCV *VP1* gene, leading to limited cross-protection among heterologous strains [8,9,10]. Despite this challenge, the efficient in vitro replication of FCV and the availability of animal models make it a valuable system for calicivirus research.

The FCV genome is approximately 7.7 kb in length and contains three open reading frames (ORFs), encoding two structural proteins and a polyprotein that is post-translationally cleaved by viral proteases into multiple non-structural proteins [11,12]. Among the structural proteins, VP2 is essential for the release of the viral genome from endosomes into the cytoplasm of infected cells [13,14], while VP1 forms the viral capsid as 90 dimers [15]. Additionally, the ORF2-encoded precursor undergoes further proteolytic processing to generate the leader of the capsid (LC) protein and mature VP1 [16]. The LC protein contributes to virus-induced apoptosis and cytopathic effects (CPEs) [17,18]. VP1 mediates viral entry by binding to the host cellular receptor junctional adhesion molecule 1 (JAM-1) and is the primary target of neutralizing antibodies, highlighting its potential as a subunit vaccine antigen [19,20,21].

In this study, an infectious clone of the FCV-GDJM202201 strain was constructed by cloning the full-length viral cDNA into the eukaryotic expression vector pcDNA3.1 under the control of the CMV promoter. Recombinant viruses, designated rGDJM-VP1_JL_ and rGDJM-VP1_SH_, were subsequently rescued by replacing the *VP1* gene of the parental strain with *VP1* genes from two heterologous FCV strains. These recombinant viruses exhibited reduced viral titers compared to the parental virus. Furthermore, neutralization assays using these recombinant viruses revealed significant differences in serum neutralizing antibody titers compared to the parental strain. Cross-neutralizing antibody assays showed that rGDJM-A4822T elicited higher neutralizing antibody titers against both homologous strain and heterologous recombinant FCVs. Collectively, this study establishes a reverse genetics system for the FCV-GDJM202201 strain and demonstrates its utility for generating VP1-substituted recombinant viruses to assess serum neutralizing antibody responses and antigenic variation.

## 2. Materials and Methods

### 2.1. Cells, Viruses, Plasmids, Reagents, and Animals

Crandell Rees feline kidney (CRFK) cells were obtained from the Wuhan Institute of Biological Products (Wuhan, Hubei, China) and cultured in Dulbecco’s Modified Eagle’s Medium (DMEM) (Gibco, Grand Island, NY, USA) supplemented with 10% fetal bovine serum (FBS) (Gibco). The FCV-GDJM202201 strain (GenBank: OQ472996.1) was isolated and stored in our laboratory. The *VP1* gene sequences of FCV-JL18 (GenBank: OR645481.1) and FCV-SH192 (GenBank: OR645482.1) were obtained from the National Center for Biotechnology Information (NCBI) database and synthesized by Sangon Biotech Co., Ltd. (Shanghai, China). The pcDNA3.1 plasmid was maintained, and mouse anti-FCV VP1 polyclonal serum was prepared in our laboratory. The ClonExpress Ultra One Step Cloning Kit V3 (C117), Phanta Flash Super-Fidelity DNA Polymerase (P521), T4 DNA ligase (C301), and Lipomaster 3000 Transfection Reagent (TL301) were purchased from Vazyme Biotech Co., Ltd. (Nanjing, Jiangsu, China). Restriction enzymes NotI (FD0593), NheI (FD0973), and ApaI (FD1414) were obtained from Thermo Fisher Scientific Inc. (Waltham, MA, USA). Fluorescein isothiocyanate (FITC)-conjugated goat anti-mouse IgG antibody (SA00003-1) was purchased from ProteinTech Group Inc. (Wuhan, Hubei, China). Feline serum samples were obtained from privately owned pet cats in Guangzhou, China, with informed consent from their owners. Blood was collected via the hindlimb vein, and serum was separated by centrifugation. Serum samples were heat-inactivated at 56 °C for 30 min. Detailed information, including breed, age, commercial names of the vaccines, and time elapsed since vaccination, is provided in the Supplementary Materials (Table S1). Specific pathogen-free (SPF) female Kunming mice (6–8 weeks old) were purchased from Zhuhai Bestest Biotech Co., Ltd. (Zhuhai, Guangdong, China) and housed at the Laboratory Animal Center of South China Agricultural University (Guangzhou, Guangdong, China) under controlled conditions, with a regular light–dark cycle and ad libitum access to food and water.

### 2.2. Construction of an Infectious Clone of FCV-GDJM202201

cDNA was synthesized from FCV-GDJM202201 RNA and used as a template to amplify the viral genome in three overlapping fragments. Specific primers and their sequences are listed in Table 1. The full-length viral genome was assembled using fusion PCR. The pcDNA3.1 vector was modified by removing the T7 promoter and inserting a hepatitis delta virus ribozyme (HDVRz) sequence downstream of the multiple cloning site. Both the modified vector and the assembled full-length viral genome were digested with NotI and ApaI, then ligated using T4 DNA ligase to generate the plasmid pFCV-GDJM202201.

CRFK cells were seeded in 6-well plates at a density of 2 × 10^5^ cells per well and cultured until reaching 70–80% confluence prior to transfection. The cells were transfected with 2.5 μg of the pFCV-GDJM202201 plasmid per well, following the manufacturer’s instructions. Cell morphology was monitored every two days, and culture supernatants from cells exhibiting CPEs were collected for immunofluorescence analysis.

### 2.3. Immunofluorescence Assay

CRFK cells were seeded into 96-well plates at a density of 1 × 10^4^ cells per well and incubated until they reached 70–80% confluence. Each well was then inoculated with 35 μL of viral supernatants and incubated at 37 °C with 5% CO_2_ for 48 h. The cells were fixed with 80% acetone at 4 °C for 30 min, followed by three washes with phosphate-buffered saline (PBS). Subsequently, the cells were incubated with 50 μL of FCV VP1 polyclonal serum (1:500 dilution) at 4 °C overnight. Finally, 50 μL of FITC-labeled goat anti-mouse IgG antibody (1:500 dilution) was added, and the plates were incubated at 37 °C for 1 h. Fluorescence was then visualized using a fluorescence microscope (AMG, Mill Creek, WA, USA).

### 2.4. Sequence Alignment and Phylogenetic Analyses of VP1

VP1 nucleotide sequences of FCV were downloaded from the NCBI database. Multiple sequence alignment was performed using the Clustal W algorithm implemented in MEGA (version 10.1.7) with default parameters. The phylogenetic tree was constructed using the maximum likelihood (ML) method based on the General Time Reversible (GTR) model with a gamma distribution and invariant sites (GTR + G + I). The robustness of the inferred tree topology was evaluated using 1000 bootstrap replicates. The phylogenetic tree was visualized and annotated using the Interactive Tree Of Life web server (https://itol.embl.de/, accessed on 9 April 2026).

### 2.5. Analysis of Viral Growth Kinetics

CRFK cells were seeded in 10 cm dishes at a density of 2 × 10^6^ cells per dish and cultured until they reached 70–80% confluence. The cells were then infected with the virus at a multiplicity of infection (MOI) of 1 or 0.01 and incubated at 37 °C for 1 h, followed by the addition of 10 mL of maintenance medium supplemented with 5% FBS to each dish. Subsequently, 200 μL of the culture supernatants were collected at the indicated time points and replaced with an equal volume of fresh maintenance medium. All collected samples were stored at −80 °C until viral titers were determined.

For viral titer determination, CRFK cells were seeded into 96-well plates. The samples were serially diluted tenfold, and 100 μL of each dilution was added to four replicate wells. The plates were incubated at 37 °C with 5% CO_2_ for 48 h, followed by an immunofluorescence assay (IFA). Viral titers were calculated as focus-forming units per milliliter (FFU/mL) using the Kärber method.

### 2.6. Virus Plaque Assay

CRFK cells were seeded into 6-well plates and incubated until reaching 80–90% confluence. The cells were then infected with FCV at an MOI of 0.001 and incubated at 37 °C with 5% CO_2_ for 1 h. Subsequently, each well was overlaid with 2 mL of a 1:1 mixture of maintenance medium containing 5% FBS and 2% low-melting-point agarose solution. The plates were incubated at 37 °C with 5% CO_2_ for 24 h. After removing the agarose overlay, the cells were washed three times with PBS and fixed with 10% formaldehyde solution for 12 h, followed by staining with 0.1% crystal violet solution for 4 h. Finally, the cells were rinsed with PBS, and the plaques were observed and imaged.

### 2.7. Neutralizing Antibody Assay

Cat sera were serially diluted threefold in 96-well plates, with two replicates per sample. Subsequently, 50 μL of 100 TCID_50_ FCV was added to each well, and the plates were incubated at 37 °C with 5% CO_2_ for 1 h. CRFK cells were then added to each well at a density of 2 × 10^4^ cells per well, followed by incubation at 37 °C with 5% CO_2_ for 48 h. IFA was performed thereafter. The neutralizing titer of the serum was defined as the highest dilution that completely inhibited viral fluorescence.

### 2.8. Immunization of Mice and Evaluation of Immunogenicity

The viral suspension (1 × 10^7.5^ FFU/mL) was inactivated with β-propiolactone and subsequently emulsified with MONTANIDE GEL adjuvant (SEPPIC, Paris, France) at a 9:1 (*v*/*v*) ratio for vaccine formulation. Twenty SPF Kunming mice were randomly divided into four groups, with 5 mice per group. Each mouse was intramuscularly immunized with 100 μL of the vaccine. A booster immunization was administered 14 days after the primary immunization, and blood samples were collected via the retro-orbital sinus 7 days post-boost. Mice in the control group were injected with 100 μL of a DMEM-adjuvant mixture at the same ratio. Serum samples were heat-inactivated at 56 °C for 30 min and subjected to neutralization assays.

### 2.9. Data Analysis

Values are expressed as the mean ± standard deviation (SD). Data analysis was performed using GraphPad Prism 8 software. Statistical significance was assessed using two-way ANOVA followed by Tukey’s multiple comparison test, with *p* values < 0.05 considered statistically significant.

## 3. Results

### 3.1. Construction of an Infectious Clone of FCV-GDJM202201

**Figure 1:**
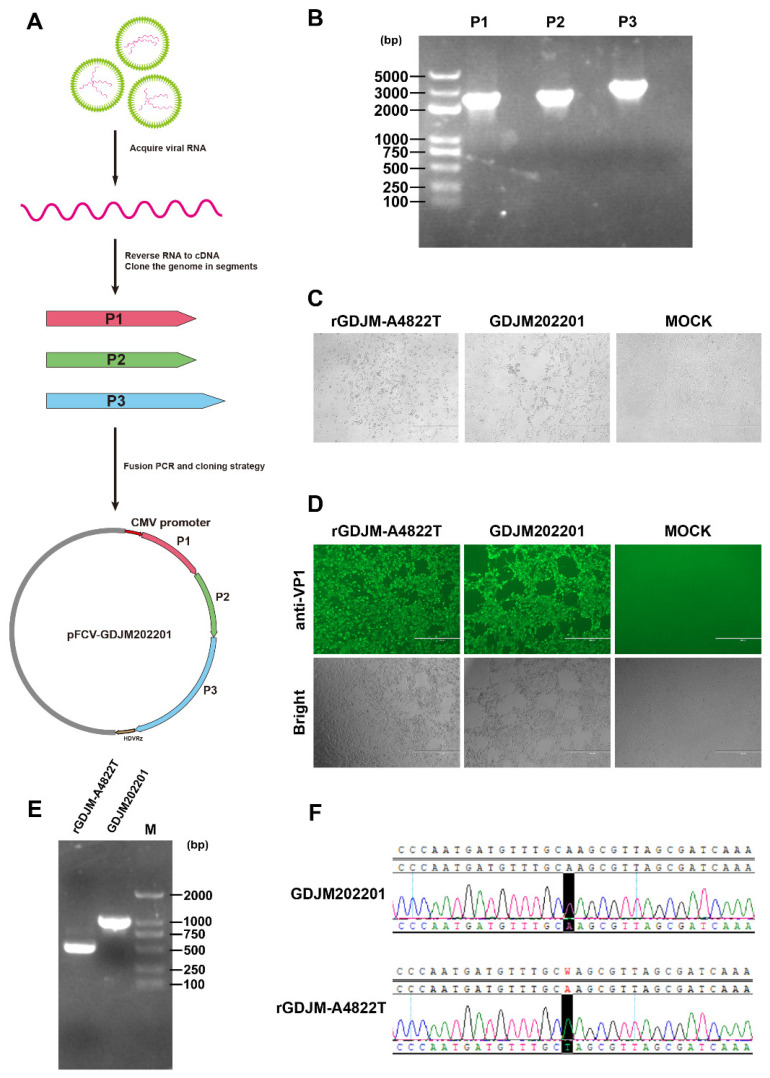
Construction of an infectious clone of FCV-GDJM202201. (**A**) Schematic illustration of the construction strategy for the pFCV-GDJM202201 plasmid. (**B**) Amplification of the viral genome as three overlapping fragments (P1–P3). (**C**) CPEs, characterized by cell rounding and aggregation, in CRFK cells following transfection with pFCV-GDJM202201. Scale bar = 400 μm. (**D**) IFA of CRFK cells infected with supernatants from CPE-positive wells, using anti-FCV VP1 polyclonal serum as the primary antibody at 2 days post-infection. Scale bar = 400 μm. (**E**) Verification of the introduced mutation at nucleotide position 4822 by NheI digestion of RT-PCR products. (**F**) Verification of the introduced mutation at nucleotide position 4822 by Sanger sequencing.

To generate the infectious clone pFCV-GDJM202201, viral RNA was extracted and reverse-transcribed into cDNA. The viral genome was amplified in three fragments and subsequently assembled by fusion PCR. The full-length viral genome was then cloned into a linearized vector to construct the plasmid pFCV-GDJM202201 (Figure 1A). The viral cDNA was amplified as three overlapping fragments with lengths of 2393 bp (P1), 2471 bp (P2), and 2941 bp (P3). A synonymous mutation was introduced at nucleotide position 4822 to create an NheI restriction site (Figure 1B). Pronounced CPEs, characterized by cell rounding and aggregation, were observed at 4 days post-transfection of pFCV-GDJM202201 in CRFK cells (Figure 1C). The supernatants from CPE-positive wells were collected and inoculated onto fresh CRFK cells, and IFA using anti-FCV VP1 polyclonal serum revealed specific green fluorescence, indicating infection by the rescued virus (Figure 1D). The rescued virus was designated rGDJM-A4822T. To verify the introduction of the engineered synonymous mutation, the corresponding genomic region of the rescued virus was amplified by RT-PCR. The PCR product derived from rGDJM-A4822T was cleaved by NheI, generating the expected restriction fragments, whereas the product from the parental virus remained undigested under the same conditions (Figure 1E). Finally, Sanger sequencing verified that the adenine at nucleotide position 4822 of rGDJM-A4822T was mutated to thymine (Figure 1F).

### 3.2. Construction of Recombinant FCVs

**Figure 2:**
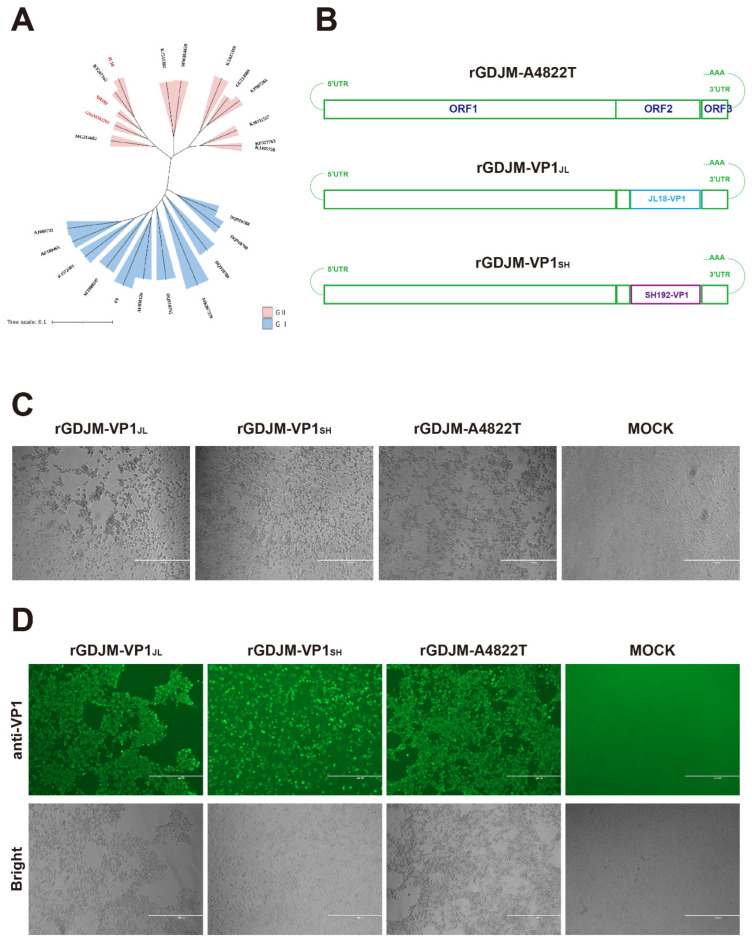
Construction and identification of recombinant FCV. (**A**) Phylogenetic tree based on *VP1* gene nucleotide sequences of FCV strains used in the present study and reference strains downloaded from the NCBI database. The evolutionary history was inferred using the ML method under the GTR + G + I model. Genogroups are indicated on the tree. The scale bar represents the number of nucleotide substitutions per site. (**B**) Schematic diagram of recombinant FCV genomes in which the *VP1* gene of pFCV-GDJM202201 was replaced with *VP1* genes from different field isolates. (**C**) CRFK cells transfected with recombinant plasmids pFCV-GDJM-VP1_JL_ and pFCV-GDJM-VP1_SH_ showing typical FCV-induced CPEs at 4 days post-transfection. Scale bar = 400 μm. (**D**) IFA of CRFK cells infected with supernatants from CPE-positive wells using anti-FCV VP1 polyclonal serum as the primary antibody at 2 days post-infection. Scale bar = 400 μm.

JL18, SH192, and GDJM202201 strains were isolated from different regions of China, including Northeast, East, and South China, respectively. Phylogenetic analysis based on the *VP1* gene showed that all three strains belonged to genogroup II (Figure 2A). The *VP1* gene of the pFCV-GDJM202201 plasmid was replaced with the *VP1* genes of JL18 and SH192, resulting in the construction of recombinant plasmids pGDJM-VP1_JL_ and pGDJM-VP1_SH_ (Figure 2B). Transfection of these recombinant plasmids into CRFK cells resulted in typical FCV-induced CPEs (Figure 2C). Supernatants from CPE-positive wells were subsequently harvested and inoculated onto fresh CRFK cells. IFA was performed using anti-FCV VP1 polyclonal serum as the primary antibody. Specific green fluorescence was observed in the infected cells (Figure 2D), confirming the successful rescue of the recombinant viruses, which were designated rGDJM-VP1_JL_ and rGDJM-VP1_SH_.

### 3.3. Growth Kinetics of Recombinant Viruses

**Figure 3:**
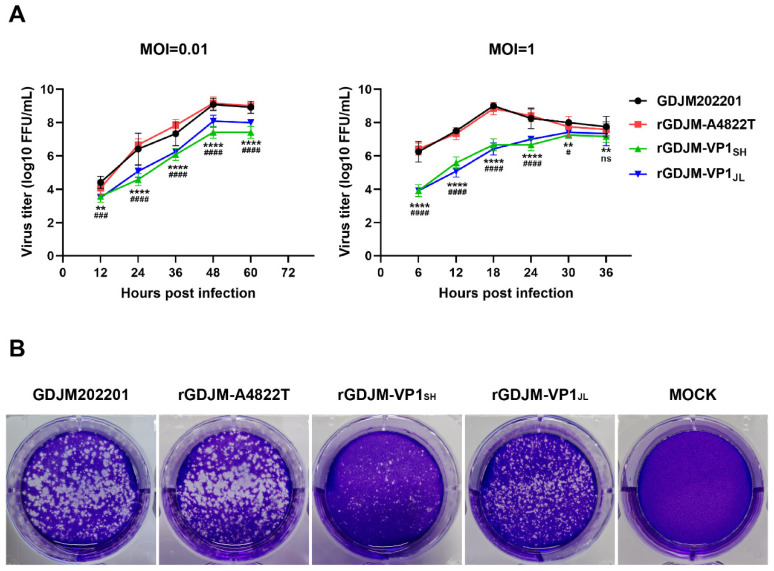
Viral growth kinetics. (**A**) CRFK cells were infected with FCV at an MOI of 0.01 or 1. Culture supernatants were collected at the indicated time points, and viral titers were determined to generate viral growth curves (*n* = 3). (**B**) CRFK cells were infected with FCV at an MOI of 0.001, and virus plaque assays were performed 24 h post-infection. Statistical analyses were performed using two-way ANOVA. # denotes significant differences between rGDJM-A4822T and rGDJM-VP1_JL_, while * denotes significant differences between rGDJM-A4822T and rGDJM-VP1_SH_ (^#^ *p* < 0.05; ** *p* < 0.01; ^###^ *p* < 0.001; ^####^ or **** *p* < 0.0001; ns, not significant).

To investigate the growth kinetics of the parental, rescued, and recombinant strains, growth curve analyses and plaque assays were performed. Both synchronized (MOI = 1) and asynchronous (MOI = 0.01) growth curves of rGDJM-A4822T were similar to those of the parental strain. Plaque assays showed that the plaque size of rGDJM-A4822T was comparable to that of the parental strain. At an MOI of 0.01, rGDJM-VP1_JL_ and rGDJM-VP1_SH_ exhibited consistently lower viral titers than rGDJM-A4822T at all time points. At an MOI of 1, both rGDJM-VP1_JL_ and rGDJM-VP1_SH_ reached peak titers later than rGDJM-A4822T. In addition, rGDJM-VP1_SH_ maintained lower titers throughout infection, whereas rGDJM-VP1_JL_ showed reduced titers at early time points but no significant difference at later time points (Figure 3A). Consistently, these recombinant viruses formed smaller plaques than rGDJM-A4822T (Figure 3B), suggesting that substitution of heterologous *VP1* genes impaired viral replication and spread in vitro.

### 3.4. Neutralizing Antibody Titers Against Recombinant FCVs in Pet Cats from Guangzhou

To evaluate the neutralizing antibody titers against the recombinant and parental viruses, serum samples were collected from pet cats in Guangzhou, China, and subjected to neutralization assays. The results showed that neutralizing antibodies against the recombinant FCVs were detectable in most cats; however, neutralizing antibody titers varied among these strains. In addition, the proportions of serum samples with neutralizing antibody titers < 1:27 against rGDJM-A4822T, rGDJM-VP1_JL_, and rGDJM-VP1_SH_ were 33.3%, 19.0%, and 4.8%, respectively, indicating low neutralizing antibody titers in a subset of cats (Table 2).

### 3.5. In Vitro Cross-Neutralizing Antibody Assay Among Recombinant FCVs

**Figure 4:**
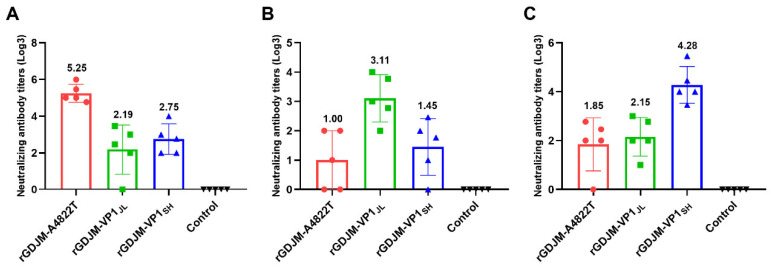
Cross-neutralizing antibody titers elicited by recombinant FCVs. (**A**–**C**) Sera collected from mice immunized with rGDJM-A4822T (**A**), rGDJM-VP1_JL_ (**B**), or rGDJM-VP1_SH_ (**C**) were evaluated for neutralizing antibody titers against homologous and heterologous recombinant viruses. Values are presented as mean ± SD.

Mice were immunized with inactivated recombinant FCVs, and serum samples were collected to evaluate cross-neutralizing antibody titers. Each recombinant FCV elicited the highest homologous neutralizing antibody titers, whereas titers against heterologous strains were reduced (Figure 4A–C). Among the recombinant FCVs, rGDJM-A4822T induced higher neutralizing antibody titers, reaching up to 1:3^5.25^, whereas rGDJM-VP1_JL_ induced lower titers, reaching up to 1:3^3.11^. Notably, sera from mice immunized with rGDJM-A4822T exhibited higher cross-neutralizing antibody titers, with titers of 1:3^2.19^ against rGDJM-VP1_JL_ and 1:3^2.75^ against rGDJM-VP1_SH_.

## 4. Discussion

Previous studies have established FCV reverse genetics systems using two primary strategies. One approach involves in vitro transcription to produce capped full-length viral genome mRNA, which is then transfected into susceptible cells to rescue infectious virus [22]. The other requires co-transfecting a plasmid containing the full-length viral genome under the control of a T7 promoter along with a T7 RNA polymerase expression plasmid, or infecting cells with a genetically modified vaccinia virus expressing T7 polymerase to generate infectious clones [23]. However, both methods are labor-intensive and costly.

To overcome these limitations, a reverse genetics system based on a eukaryotic expression vector driven by the human elongation factor-1α (EF-1α) promoter was subsequently developed, enabling the direct rescue of infectious virus through plasmid transfection into susceptible cells [24,25]. In the present study, we constructed an infectious clone of the FCV-GDJM202201 strain using the eukaryotic expression vector pcDNA3.1, in which viral genome transcription is driven by the CMV promoter. Similar to the EF-1α promoter-based system, infectious FCV was successfully rescued following transfection of susceptible cells with the full-length viral genome plasmid.

Previous studies using the FCV reverse genetics system have demonstrated that mutation of the tyrosine residue at position 24 of the viral protein genome-linked (VPg) abolishes the recovery of infectious virus [26]. This impairment is presumably due to the inability of the mutated tyrosine to mediate the covalent linkage between VPg and the viral genome [27]. In contrast, mutations or deletions in VP2 do not affect viral genome replication but impair the assembly of infectious virions [13]. Additionally, the 3′ untranslated region of the FCV genome has been shown to be essential for viral replication, while the length of the polyadenylate tail is critical for successful viral rescue [24].

Beyond functional analysis, the FCV reverse genetics system also serves as a valuable tool for constructing replication-deficient strains with potential applications as vaccine candidates [28]. In the present study, replacing the *VP1* gene of the GDJM202201 strain with that of the JL18 or SH192 strains resulted in significantly altered viral growth kinetics. The recombinant viruses rGDJM-VP1_JL_ and rGDJM-VP1_SH_ exhibited significantly reduced viral titers compared with the parental strain, likely due to decreased efficiency of viral adsorption and entry following *VP1* substitution. Consistent with our findings, previous studies have reported that virulent FCV replicates more rapidly than less virulent ones [5,29], and that in vitro replication efficiency is closely associated with the *VP1* gene [30].

FCV exhibits substantial strain diversity, with genetic variation in the *VP1* gene leading to limited cross-protection among heterologous strains. To address this challenge, the present study proposes a strategy for constructing recombinant FCV harboring distinct *VP1* genes using a reverse genetics system. These recombinant viruses provide a robust and genetically controlled platform for evaluating the cross-protective efficacy of neutralizing antibodies, enabling direct assessment of antigenic differences attributable specifically to *VP1* variation. Compared to conventional strain isolation, this approach significantly reduces the time required and overcomes geographical constraints on strain availability. Consequently, this system facilitates more precise evaluation of antigenicity and vaccine-related immune responses, thereby supporting vaccine development and the assessment of immunogenicity.

## 5. Conclusions

In summary, we successfully established an infectious clone of the FCV-GDJM202201 strain, constructed recombinant viruses carrying *VP1* genes from heterologous FCV strains, and used these recombinant viruses to evaluate serum neutralizing antibody responses. Neutralization assays demonstrated variation in serum neutralizing capacity among recombinant FCVs, with rGDJM-A4822T exhibiting higher neutralizing antibody titers and cross-neutralizing activity.

## Figures and Tables

**Table 1:** Primers used in this study.

Primer	Primer Sequence (5′-3′)
F-P1	ATAAGAAT*GCGGCCGC*GTAAAAGAATTTTGAG
R-P1	TGAAGTGGCAAAGATGGGTGAGATCGG
F-P2	CACCCATCTTTGCCACTTCATAAAAGG
R-P2	CGCTAACGCTAGCAAACATCATTGGG
F-P3	GATGTTTGCTAGCGTTAGCGATC
R-P3	CT*GGGCCC*TTTTTTTTTTTTTTTTTTTTTTTTTTTTTTTTTTTTTTCCCTGGGGTTAGG

The part in italics in the primer sequence is the restriction site.

**Table 2:** Neutralization assays. Sera were collected from cats and tested for neutralizing antibodies against the indicated viral strains. Neutralizing antibody titer (1:x).

Cat	1	2	3	4	5	6	7
rGDJM-VP1_SH_	27	81	243	45	81	81	729
rGDJM-VP1_JL_	243	81	243	27	243	27	729
rGDJM-A4822T	189	27	81	27	27	<27	729
Cat	8	9	10	11	12	13	14
rGDJM-VP1_SH_	729	243	<27	405	243	81	405
rGDJM-VP1_JL_	729	63	<27	243	243	27	243
rGDJM-A4822T	243	<27	<27	81	135	<27	243
Cat	15	16	17	18	19	20	21
rGDJM-VP1_SH_	243	27	243	63	45	81	729
rGDJM-VP1_JL_	567	<27	243	<27	<27	243	729
rGDJM-A4822T	243	<27	81	<27	<27	45	567

## Data Availability

The data supporting the findings of this study are available from the corresponding author upon reasonable request, subject to institutional and ethical regulations.

## Supplementary Materials

The following supporting information can be downloaded at: https://www.mdpi.com/article/10.3390/ani16081237/s1. Table S1: Detailed information on the cats used in this study, including breed, age, commercial names of the vaccines, and time elapsed since vaccination.
